# Four Hour Temporal Relation of 5-HTP and L-DOPA Induces Inhibitory Responses in Recrudescing Gonad of Indian Palm Squirrel (*Funambulus pennantii*)

**DOI:** 10.1155/2013/206876

**Published:** 2013-07-01

**Authors:** Ranjana Jaiwal, C. M. Chaturvedi

**Affiliations:** ^1^Department of Zoology, M. D. University, Rohtak 124001, India; ^2^Department of Zoology, Banaras Hindu University, Varanasi 221005, India

## Abstract

Daily injections of L-dihydroxyphenylalanine (L-DOPA, dopamine precursor) given 4 h after 5-hydroxytryptophan (5-HTP, serotonin precursor) induced inhibitory responses in recrudescing gonad (in the first week of December) of Indian palm squirrel, a seasonally breeding subtropical animal. Other temporal relations (L-DOPA given at 0, 8, 12, 16, and 20 h after 5-HTP administration) did not show any effect on the recrudescing gonad. This inhibitory effect of 4 h was evident under short day length (6 : 18) group but was masked by the increasing day length of nature (NDL, late December onwards) and increased photoperiod of long day group (16 : 8). It is apparent that seasonal testicular recrudescence of Indian palm squirrel during short day length by 4 h relation of 5-HTP and L-DOPA is not a pharmacological effect but actually is an alteration of seasonality in this annually breeding mammal. It seems that endogenous mechanism controlling seasonal testicular recrudescence of Indian palm squirrel is reset by timed daily injections of these neurotransmitter drugs. It is suggested that in spite of different environmental factors (photoperiod, humidity, etc.) used by different species to time their annual reproduction, basic mechanism of seasonality appears to be the same, that is, the temporal synergism of neurotransmitter activity.

## 1. Introduction

Injections of two neurotransmitter precursor drugs the 5-hydroxytryptophan (5-HTP, serotonin precursor) and L-dihydroxyphenylalanine (L-DOPA, dopamine precursor) are thought to reset the phase of two circadian neuroendocrine oscillations with serotonergic and dopaminergic components and thereby determine seasonality [1]. A mechanism for neuroendocrine regulation of seasonality was proposed in different species of animals like a teleost fish [2], white throated sparrow [3], Japanese quail [4, 5], spotted munia [6, 7], red headed bunting [8], lal munia [9], hamster [10], Indian palm squirrel [11, 12], Indian weaver bird [13], Indian garden lizard [14], and so forth. Temporal synergism of 5-HTP and L-DOPA was not only found to be gonadostimulatory by 12 h relationship in squirrel [11] but also the advancement of puberty in Japanese quail [15]. On the other hand, the 8 h relation inhibits gonadal growth and sometimes induces gonadal suppression [16]. Recently, Sethi and Chaturvedi [17] had reported that in mice 8 h temporal relation of 5-HTP and L-DOPA suppresses gonadal growth and 12 h relation accelerates gonadal growth, whereas other relations (0, 4, 16, and 20 h) were ineffective. Further, the inhibitory effects of an 8-h phase relation and the stimulatory effects of a 12-h phase relation of neural oscillations on the gonadal growth of mice are inversely correlated with levels of RFamide related peptide-3 (RFRP-3), a mammalian ortholog of avian gonadotropin-inhibitory hormone [18]. The stimulatory relationship of 12 h completely eliminated seasonal reproductive regressions and animals were maintained in full breeding condition unlike control where total gonadal quiescence was achieved [12]. Chaturvedi and her group suggest that seasonally breeding species have daily rhythms in the secretion of hormones (corticosterone and prolactin) and neurotransmitters (serotonin and dopamine), and when these (hormones and neurotransmitters) exist in a particular phase relationship to each other, this phase relation initiates or terminates seasonal breeding and related events. Moreover, a different phase relationship has been reported in the circadian hypothalamic serotonin and dopamine rhythms of breeding versus nonbreeding quail under both control and simulated conditions [19, 20] and in spawning and nonspawning fish [21]. In view of these previous studies, present long term study was conducted in recrudescing/preparatory phase of annual gonadal cycle to study the interaction, if any, between the external (photoperiod) and internal (neuroendocrine entrainment by neurotransmitter precursor drug) factors. Since 4 h relation is thought to be gonadosupressive, its effect was determined during the period when initiation of seasonal gonadal growth takes place (in first week of December) and continued up to fully active reproductive phase (June) to observe the effect of neurotransmitter drugs.

## 2. Materials and Methods

Adult Indian palm squirrels (body weight: 120 ± 5 g) were kept in wire net cages in a room fully exposed to ambient environmental conditions. The study was conducted in the recrudescing phase (December with L : D 10 : 14 h, temp 10–15 ± 5°C, relative humidity 70 ± 5%) of annual gonadal cycle. The animals were acclimatized to laboratory conditions and fed with soaked gram (*Cicer arietinum*)seeds and water *ad libitum*. Thirty-six squirrels were divided into two groups. Group I received two daily injections (0.1 mL intra-peritoneally) of normal saline at an interval of 4 h (i.e., at 8 a.m. and 12 p.m.), and Group II received daily injections of 5-HTP at 8 a.m. and L-DOPA at 12 p.m. The precursor drugs for serotonin and dopamine, 5-HTP and L-DOPA (5 mg/100 g body weight), were administered intraperitoneally in 0.1 mL solution of normal saline for a period of 11 days. These doses of 5-HTP and L-DOPA are reported to increase the brain content of serotonin [22–24] and dopamine [25, 26], respectively, in rats. To avoid possible photoperiodic interference with neuroendocrine entrainment by neurotransmitter precursor injections, squirrels were maintained under continuous condition of light (LL, 300 lux) during the period of treatment for 11 days. Testicular measurements were taken before (initial) and after treatment (0 week following treatment). Following treatment, each group was further divided into subgroups according to Table 3, and the experiment was continued up to June.

Repeated measurements of testicular volume were taken at 0, 2, 4, 6, 8, 15, 17, 21, and 25 weeks. Finally, after 25th week of treatment, animals were weighed and sacrificed by decapitation; testes and accessory sex organs were removed, weighed, and fixed in Bouin's fluid for histological studies. The volume of the testes was calculated using the formula 4/3 *π*a*b* ^2^ where *a* = 1/2 of the long axis and *b* = 1/2 of the short axis. Statistical analysis was done by using, *t*-test, ANOVA, and Newman keul's multiple range test [27, 28].

## 3. Results

### 3.1. Normal Day Light

**Figure 1:**
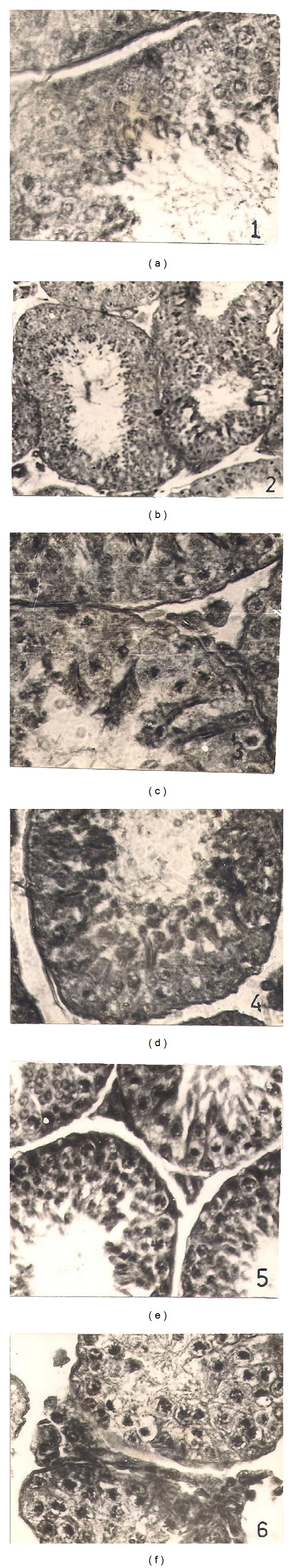
(a) T. S. of testis of NDL—control squirrel showing active condition. (b) T. S. of testis of NDL—4 h squirrel showing active condition. (c) T. S. of testis of LD 16 : 8—control squirrel showing full breeding condition. Bunched spermatozoa can be seen. (d) T. S. of testis of LD 16 : 8—4 h squirrel showing active condition. (e) T. S. of testis of LD 6: 18—control squirrel showing active condition. (f) T. S. of testis of LD 6 : 18—4 h squirrel showing inactive condition.

When compared to initial values, final testicular volume of squirrels of control as well as experimental subgroup increased significantly (Table 1). But when final value of NDL control and experimental squirrel were compared, testicular volume (Table 1), gonadosomatic index (GSI), and weight of accessory sex organs of 4 h treated squirrels were significantly less (Table 2). Histologically, testis of control squirrel showed full breeding condition with active spermatogenesis, while that of experimental group showed moderate activity but could not reach up to the extent of control testis (Figures 1(a) and 1(b)).

### 3.2. Light : Dark (16 : 8)

A significant increase was also observed in testicular volume of both the subgroups (control 383% and experimental 310%) during long day treatment, when compared to their initial size (Table 1). A similar trend was also observed in gonadosomatic index (GSI) and weight of accessory sex organs of control and treated group (Table 2). Histologically, also both groups showed full breeding condition (Figures 1(c) and 1(d)).

### 3.3. Light : Dark (6 : 18)

 When compared to initial size a significant increase (90%) was noted in the testicular volume of control, but that of 4 h treated group decreased significantly (−42%) (Table 2). At the termination of the study, testicular volume (Table 1), gonadosomatic index (GSI), and weight of accessory sex organs of 4 h treated squirrels were significantly less compared to their controls (Table 2). Histologically, while control testis had progressive development with smaller seminiferous tubules, 4 h treated testis showed fully regressed condition with thick tunica albuginea and very small seminiferous tubules having inactive spermatogonial cells (Figures 1(e) and 1(f)).

## 4. Discussion

 Seasonal gonadal recrudescence of Indian palm squirrel starts from approximately mid-December, attaining full breeding condition by January which is maintained till June/July [11]. Results indicate that daily administration of dopaminergic precursor drug (L-DOPA) given 4 h after serotonergic precursor drug (5-HTP) induce inhibiting responses in sexually recrudescing testis unlike control squirrels. Control squirrels maintained under NDL and long days followed the same pattern, and peak values were noted at the termination of the study (in June) leading to full gonadal growth unlike LD 6 : 18. Testicular volume of control group exhibit 383%, and 365% increase under long days and NDL, respectively, only 90% increase was observed in short day group (Table 1). Further, by 4 h temporal relation of the two precursor drugs, testicular volume also increased in long day (310%) and NDL (130%) group, but a decrease (−42%) was observed in short day group when compared to their initial values (Table 1). The similar pattern was observed in the accessory sex organs and gonadosomatic index (Table 2). It is obvious that although rate and degree of development of control groups was different in the three photo regimes, the seasonal gonadal growth was not interrupted by any day length. It may be suggested that endogenous mechanism initiating and maintaining seasonal gonadal growth at this phase of the annual cycle is operative in all the three-day lengths; however, rate of gonadal development depends on the availability of daily photoperiod.

 On the basis of partial gonadal development under short day length it may be suggested that squirrels were certainly not scotosensitive in this phase, although complete scotorefractoriness could not be achieved. However, scotosensitive response was observed in those short day squirrels which received daily injections of 5-HTP and L-DOPA in 4 h temporal relationship. The 4 h relation of serotonergic and dopaminergic activity inhibit/suppress gonadal activity irrespective of available day length. Degree of suppression was in accordance with the stimulatory effect of day length. This dual mechanism that is, stimulation by longer day length and inhibition by 4 h temporal relation of serotonergic and dopaminergic activity allowed maximum gonadal growth under long days and minimum growth under short days. It seems that the gonadoinhibitory effect of 4 h relation has overpowered the stimulatory effect of long days. Further it has also suppressed the gonadal development in natural day length compared to its control. Moreover the inhibitory effect of short day length was further attenuated by 4 h phase relation. Obviously the 4 h has induced gonado inhibitory effect irrespective of the photoperiod in which the animal is kept. It also interferes with the inhibitory effect of the short day length.

The endogenous mechanism controlling seasonal reproduction during breeding phase, regressive phase and recrudescing phase, in Indian palm squirrel is apparently reset by timed daily injections of 5-HTP and L-DOPA. The 12 h temporal relation of drug treatment not only maintained the breeding condition by eliminating seasonal gonadal regression but also restimulated the regressing gonad to full development [11, 12], and 8 h suppresses the annual testicular development [16]. The 4 h treatment under short day inhibits the recrudescing testis (present study), and this inhibitory effect of 4 h was obvious but masked by the increasing day length of nature (NDL group), and increased photoperiod of long day (LD 16 : 8) group (Figure 1). 

It may be suggested that apparent seasonal testicular recrudescence of Indian palm squirrel during short day length by 4 h relation of 5-HTP and L-DOPA is not a pharmacological effect but actually is an alteration of seasonality in this annually breeding mammal. It seems that endogenous mechanism controlling seasonal testicular recrudescence of Indian palm squirrel is reset by timed daily injections of these neurotransmitter drugs. It is also suggested that in spite of different environmental factors (photoperiod, humidity, etc.) used by different species to time their annual reproduction, basic mechanism of seasonality appears to be the same, that is, the temporal synergism of neurotransmitter activity.

## Figures and Tables

**Table 1:** Testicular volume of Indian palm squirrel given timed daily injections of 5-HTP and L-DOPA (4 h relation) in different photoperiodic schedules (LD 6 : 18; NDL; LD 16 : 8).

Photoperiodic schedule	Treatment	Initial	Final^a^	% change from initial
LD 6 : 18	Control	0.1878 ± 0.062	0.3629 ± 0.008^b∗^	+90%
	4 h	0.1824 ± 0.074	0.1576 ± 0.0036^c∗^	−42%
NDL	Control	0.1850 ± 0.05	0.9011 ± 0.021**	+365%
	4 h	0.1841 ± 0.001	0.4603 ± 0.0082**	+130%
LD 16 : 8	Control	0.1798 ± 0.08	0.9369 ± 0.28*	+383%
	4 h	0.1801 ± 0.027	0.6818 ± 0.0077*	+310%

^a^All the groups differ significantly, *P* < 0.001 (ANOVA).

^b^Differ significantly from final untreated controls of NDL and LD 16 : 8 (*P* < 0.05, multiple range test).

^c^Differ significantly from other groups (*P* < 0.05, multiple range test).

Significance of difference from respective initial control (*t*-test).

*P < 0.01; **P < 0.001.

**Table 2:** Gonadosomatic index (GSI) and weight of accessory sex organs of Indian palm squirrel given timed daily injections of 5-HTP and L-DOPA (4 h relation) in different photoperiodic schedules (LD 6 : 18; NDL; LD 16 : 8).

mg organ wt./100 gm body wt.	LD 6 : 18		NDL		LD 16 : 8	
	Control	4 h	Control	4 h	Control	4 h
Gonadosomatic index	380 ± 38	200 ± 29*	912 ± 39	590 ± 50*	1015 ± 58	825 ± 65
Seminal vesicle	76 ± 27	56 ± 18	235 ± 18	146 ± 20*	227 ± 20	216 ± 20
Prostate gland	78 ± 26	60 ± 20	285 ± 37	164 ± 29*	236 ± 37	230 ± 29

Values are mean ± SE.

Significance of difference from respective control *P < 0.001.

**Table 3:** Diagrammatic representation of experimental procedure.

Group	Control (C)	Experimental (E)
*Treatment *		
(Under continuous light (LL) for 11 days in December)	Normal saline	4 h relation of 5-HTP and L-DOPA
*Post treatment *		
(Shifted to different photoperiods)	C_ 1 _ —Light : Dark (6 : 18)—E_1_	
	C_2_—Natural Day Light—E_2_	
	C_3_—Light : Dark (16 : 8)—E_3_	
Termination	After 25 weeks (in June)	
